# Theme Trends and Knowledge Structure on Mobile Health Apps: Bibliometric Analysis

**DOI:** 10.2196/18212

**Published:** 2020-07-27

**Authors:** Cheng Peng, Miao He, Sarah L Cutrona, Catarina I Kiefe, Feifan Liu, Zhongqing Wang

**Affiliations:** 1 Department of Ophthalmology The Fourth Affiliated Hospital of China Medical University Shenyang China; 2 Department of Information Center The First Hospital of China Medical University Shenyang China; 3 Department of Population and Quantitative Health Sciences University of Massachusetts Medical School Worcester, MA United States; 4 Center for Healthcare Organization and Implementation Research Edith Nourse Rogers Memorial Veterans Hospital Bedford, MA United States

**Keywords:** mobile app, mobile health, mhealth, digital health, digital medicine, bibliometrics, co-word analysis, mobile phone, VOSviewer

## Abstract

**Background:**

Due to the widespread and unprecedented popularity of mobile phones, the use of digital medicine and mobile health apps has seen significant growth. Mobile health apps have tremendous potential for monitoring and treating diseases, improving patient care, and promoting health.

**Objective:**

This paper aims to explore research trends, coauthorship networks, and the research hot spots of mobile health app research.

**Methods:**

Publications related to mobile health apps were retrieved and extracted from the Web of Science database with no language restrictions. Bibliographic Item Co-Occurrence Matrix Builder was employed to extract bibliographic information (publication year and journal source) and perform a descriptive analysis. We then used the VOSviewer (Leiden University) tool to construct and visualize the co-occurrence networks of researchers, research institutions, countries/regions, citations, and keywords.

**Results:**

We retrieved 2802 research papers on mobile health apps published from 2000 to 2019. The number of annual publications increased over the past 19 years. *JMIR mHealth and uHealth* (323/2802, 11.53%), *Journal of Medical Internet Research* (106/2802, 3.78%), and *JMIR Research Protocols* (82/2802, 2.93%) were the most common journals for these publications. The United States (1186/2802, 42.33%), England (235/2802, 8.39%), Australia (215/2802, 7.67%), and Canada (112/2802, 4.00%) were the most productive countries of origin. The University of California San Francisco, the University of Washington, and the University of Toronto were the most productive institutions. As for the authors’ contributions, Schnall R, Kuhn E, Lopez-Coronado M, and Kim J were the most active researchers. The co-occurrence cluster analysis of the top 100 keywords forms 5 clusters: (1) the technology and system development of mobile health apps; (2) mobile health apps for mental health; (3) mobile health apps in telemedicine, chronic disease, and medication adherence management; (4) mobile health apps in health behavior and health promotion; and (5) mobile health apps in disease prevention via the internet.

**Conclusions:**

We summarize the recent advances in mobile health app research and shed light on their research frontier, trends, and hot topics through bibliometric analysis and network visualization. These findings may provide valuable guidance on future research directions and perspectives in this rapidly developing field.

## Introduction

Worldwide, the use of mobile phones has reached widespread popularity at an unprecedented rate [1]. There were more than 325,000 mobile health apps in 2017 [2]. According to Zion Market Research, the global mobile health app market will grow from $8.0 billion in 2018 to $111.1 billion by 2025 [3]. Recently, mobile health apps have seen significant growth. An increasing number of hospitals and health care institutions are using mobile health apps to monitor the development of diseases and improve health care outcomes [4]. Therefore, it is essential to understand the use, significance, trends, and research hot spots of mobile health apps in the health care domain.

Bibliometric analysis has been widely used in quantitative analysis of academic literature to describe the hot spots, trends, and contributions of scholars, journals, and countries/regions [5-8]. Co-word analysis, proposed in the late 1970s [9,10] as an important bibliometric technique, can identify the main themes, investigate hot spots, and detect knowledge in literature. Thus, bibliometrics can contribute to monitoring the development and patterns of effective publications [11]. In recent years, bibliometric analysis has been applied to biomedicine and health care [12-15]. In the current study, we used bibliometric quantitative analysis and network visualization to describe the research trends, research hot spots, emerging topics, and collaboration partners in the field of mobile health apps. Our study is the first one to quantitatively analyze the characteristics and hot topics of mobile health app research. Our study may provide valuable guidance on future research directions in this rapidly developing field.

## Methods

### Data Collection

Web of Science (WOS) is an extensive international database of academic information, including more than 9000 prestigious and high-impact research journals from all over the world. WOS contains various characteristics that can be used for bibliometric study, including title, author, institution, country/region, publication year, and keywords [9]. WOS has been recently receiving more attention as a reliable data source for bibliometric analysis in the biomedical domain, with applications in clinical and bench science research questions (eg, cardiovascular disease, diabetic kidney disease, and long noncoding RNA) [8,16,17]. On October 6, 2019, we conducted a publication search in WOS to find publications using the following search strategy: TS=“mobile health app*” OR (TS=“mobile app*” AND TS=(“health*” OR “medic*” OR “clinic*” OR “hospital*”)). Only full-length papers were included, and no language limitation was set. We validated the reliability of our search strategy by manually reviewing the retrieved publications [18]. All data from retrieved publications were collected and saved in TXT formats.

### Data Analysis and Visualization Maps

We aimed to exploit bibliometric analysis to identify the knowledge structure, research frontiers, research hot spots, active authors, and other bibliometric information in the mobile health app area. Bibliometric analysis typically consists of the construction of bibliometric maps and the graphical representation of such maps [19]. Co-word analysis was used to calculate the frequency of co-occurrence of bibliographic information and perform hierarchical clustering based on the co-occurrence information [9,10]. Finally, the clusters were visualized graphically.

In this study, we have applied widely used bibliometric analysis tools on the WOS data. Bibliographic Item Co-Occurrence Matrix Builder version 2.0 [10] was used to extract and analyze bibliographic information on the publication years and the journal sources. VOSviewer (version 1.6.13; Leiden University) was used to extract bibliographic information on researchers, institutions, countries/regions, references, and keywords. VOSviewer uses the visualization of similarities mapping technique, which produces better structured maps than other popular multidimensional scaling techniques for bibliometrics [19]. Specifically, when constructing a map, VOSviewer takes as input a similarity matrix that is created using a similarity measure known as the association strength [20]. It calculates the similarity *s_ij_* of two items *i* and *j* with the equation *s_ij_* = *c_ij_* / *(w_i_w_j_)*, where *c_ij_* denotes the number of co-occurrences of items *i* and *j*, and where *w_i_* and *w_j_* denote the total number of occurrences of items *i* and *j*. Once the similarity matrix is created, VOSviewer maps all the items in a 2-dimensional map so that items with a high similarity will be located close to each other, while items with a low similarity will be located far from each other. Unlike other map-viewing programs, VOSviewer pays special attention to the graphical representation of bibliometric maps in an easy-to-interpret way [19].

Using network-mapping techniques, we created different bibliometric maps that included coauthorships of authors, institutions, and countries/regions; co-citations of references; and co-occurrence of keywords. Each node in a map is represented by a circle with a label. Larger circles indicate higher-frequency items. The color of each circle is determined by the clusters it belongs to. The thickness and length of links between nodes represent the association strength between corresponding nodes. A maximum of 500 lines was set to display the 500 strongest links between nodes.

### Research Ethics

Data from bibliographic information were searched and downloaded from WOS. These were publicly available data. The extraction of these data did not involve interaction with human subjects or animals. Thus, there were no ethical issues involving the use of these data, and no approval from an ethics committee was required.

## Results

### Publication Outputs

Based on our search strategy, we identified and incorporated 2802 publications on mobile health apps from WOS. The number of annual publications on mobile health apps increased from 2 publications in 2000 to 692 publications in 2018 (2019 data are incomplete because they reflect only approximately 9 months of publications). Before 2013, the number of annual publications did not exceed 100. However, the number of annual publications in 2014, 2015, 2016, 2017, and 2018 was 122, 263, 430, 507, and 692, respectively.

### Distribution of Source Journals

Publications on mobile health app research were distributed across 1209 journals; 848 of these journals have published only 1 paper on mobile health apps. Table 1 lists the top 10 journals on this topic. *JMIR mHealth and uHealth* published the most papers (323/2802, 11.53%), followed by *Journal of Medical Internet Research* (106/2802, 3.78%), then *JMIR Research Protocols* (82/2802, 2.93%). The top 10 journals published 776 publications, accounting for 27.64% of all publications in this study.

**Table 1:** Top 10 journals publishing research on mobile health app research, 2000-2019.

Rank	Journal	Country	Categories	Publications, n	Percentage^a^
1	*JMIR mHealth and uHealth*	Canada	Medical informatics	323	11.53
2	*Journal of Medical Internet Research*	Canada	Medical informatics	106	3.78
3	*JMIR Research Protocols*	Canada	Medical informatics	82	2.93
4	*Plos One*	United States	Multidisciplinary sciences	47	1.68
5	*Journal of Medical Systems*	United States	Medical informatics	43	1.53
6	*BMC Medical Informatics and Decision Making*	England	Medical informatics	37	1.32
7	*International Journal of Medical Informatics*	Ireland	Medical informatics	37	1.32
8	*Telemedicine and e-Health*	United States	Health care sciences and services	37	1.32
9	*JMIR Mental Health*	Canada	Medical informatics and mental health	33	1.18
10	*BMJ Open*	England	Medicine, general and internal	31	1.11

^a^The total number of retrieved papers on mobile health apps from 2000 to 2019 (N=2802) was used as the denominator.

### Distribution and Coauthorship of Countries/Regions

**Figure 1:**
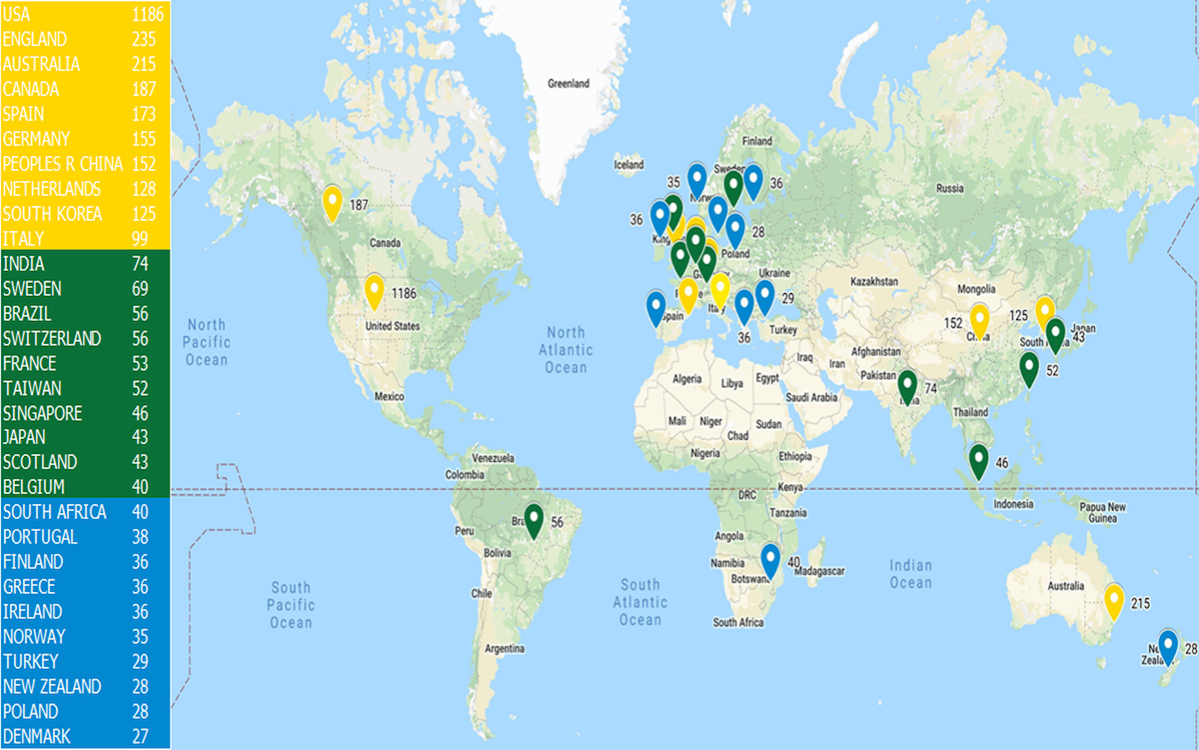
Top 30 countries/regions publishing mobile health app research, 2000-2019.

According to the search results, 2802 publications came from 104 countries/regions. Figure 1 shows the location of the top 30 countries/regions that were publishing mobile health app research. The United States has the largest number of publications (1186/2802, 42.33%) and England ranks second (235/2802, 8.39%), followed by Australia (215/2802, 7.67%) and Canada (112/2802, 4.00%).

**Figure 2:**
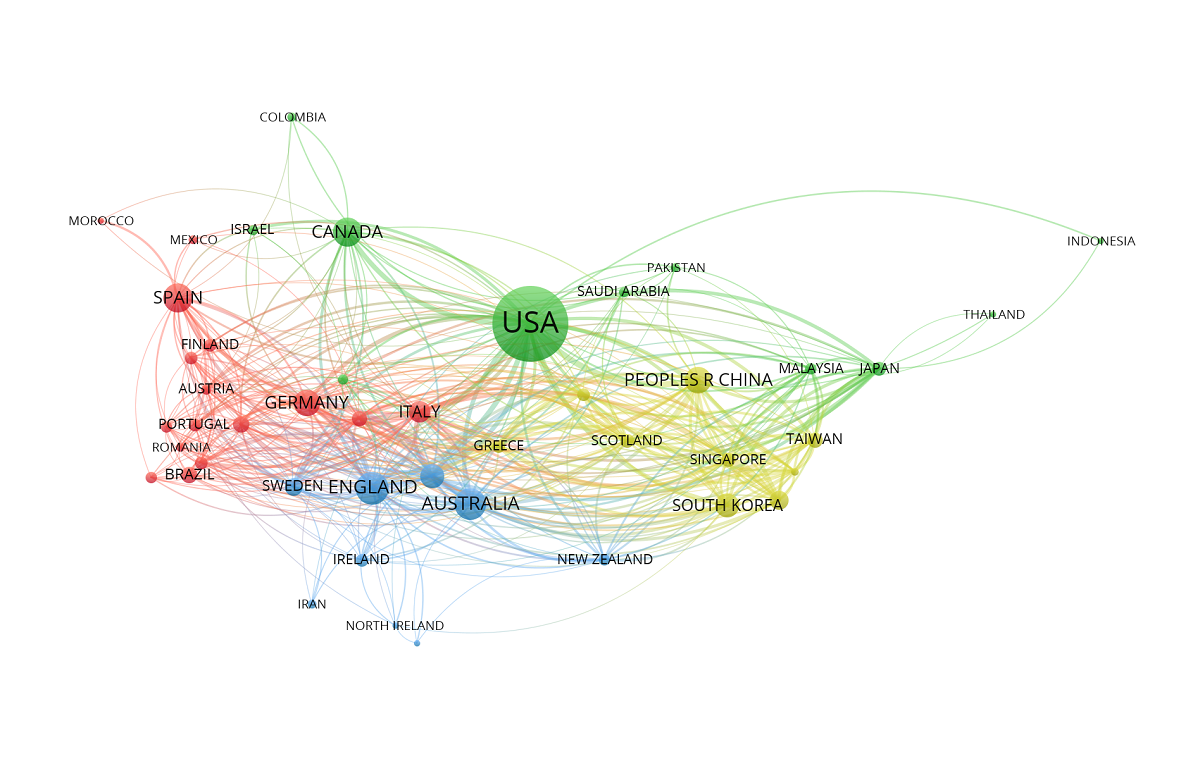
The coauthorship network of countries/regions that contributed to mobile health app research, 2000-2019. Peoples R China: People's Republic of China. USA: United States of America.

As shown in Figure 2, the coauthorship analysis of countries/regions reflects the collaboration relationship between countries/regions in this field, as well as the degree of collaboration. The larger nodes represent more productive countries/regions in this field; the thickness and length of links between nodes represent the cooperative relationship between countries/regions. Figure 2 shows the 45 most productive countries/regions in this field from 4 collaboration clusters, which were distinguished by different colors.

### Distribution and Coauthorship of Institutions

According to the search results, 3795 research institutions contributed to mobile health app research. Table 2 presents the top 10 most productive institutions in mobile health app research. The University of California San Francisco (67 publications) ranked first among all institutions identified, followed by the University of Washington (58 publications) and the University of Toronto (56 publications).

**Table 2:** Top 10 most productive institutions in mobile health app research, 2000-2019.

Rank	Institution	Country	Publications, n	Citations, n
1	Univ^a^ of California San Francisco	United States	67	819
2	Univ of Washington	United States	58	511
3	Univ of Toronto	Canada	56	640
4	Stanford Univ	United States	46	432
5	Univ of Pittsburgh	United States	45	409
6	Harvard Medical School	United States	42	408
7	Columbia Univ	United States	39	387
8	Northwestern Univ	United States	39	296
9	Univ of Sydney	Australia	39	240
10	Seoul National Univ	South Korea	33	184

^a^Univ: university.

**Figure 3:**
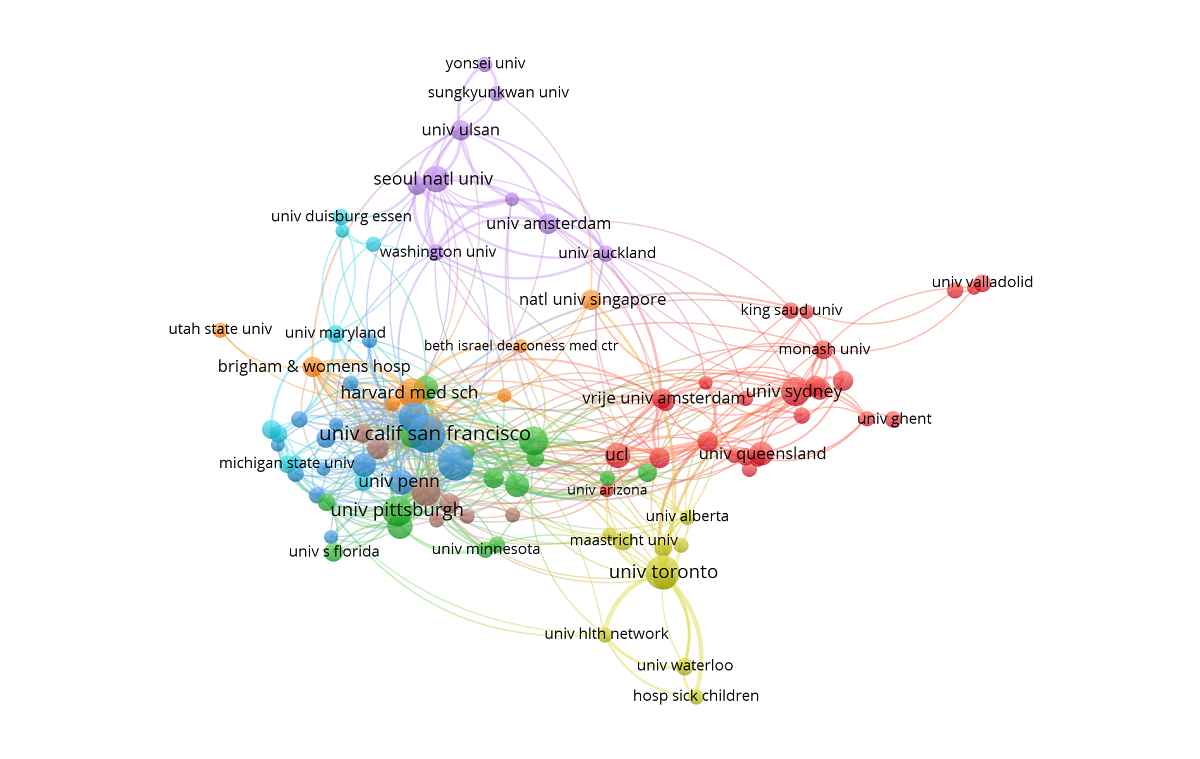
The coauthorship network of institutions that contributed to mobile health app research, 2000-2019. Univ: university.

Coauthorship analysis was performed by VOSviewer to display the visualization network map of institutions in mobile health app research. The link between institutions is determined by the number of publications coauthored between them. The coauthorship analysis of institutions shows that 99 institutions, each of which published at least 10 papers, formed 8 clusters. These clusters are shown in Figure 3 and distinguished by different colors.

### Distribution and Coauthorship of Authors

According to the search results, 2802 mobile health app publications were written by 13,040 authors, with an average of 5 authors per publication. Table 3 presents the top 10 most productive authors (all authors of each publication were ranked equally) in mobile health app research. Schnall R (15 publications) ranked first among all authors, followed by Kuhn E (14 publications), Lopez-Coronado M (14 publications), and Kim J (14 publications).

**Table 3:** Top 10 most productive authors in mobile health app research, 2000-2019.

Rank	Author	Publications, n	Citations, n
1	Schnall R	15	217
2	Kuhn E	14	223
3	Lopez-Coronado M	14	130
4	Kim J	14	43
5	Lee S	13	365
6	Li J	13	49
7	Torous J	12	271
8	Lee JH	10	107
9	Lee J	10	46
10	Zhang Y	10	36

**Figure 4:**
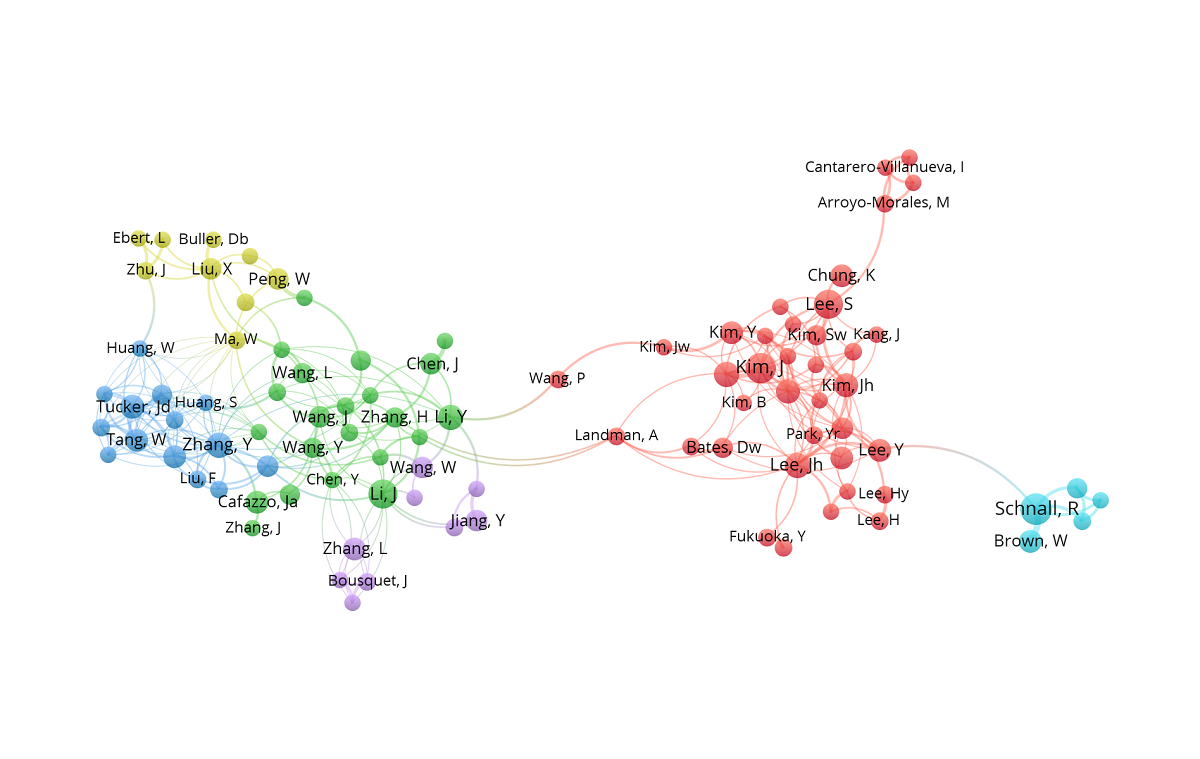
The coauthorship network of authors who contributed to mobile health app research, 2000-2019.

Our coauthorship analysis of authors showed that 221 of 13,040 authors had published at least 4 papers, and the largest set of associated authors consisted of 95 authors in 6 clusters. The node label shows the author's name, and the node size represents the number of published publications. Links connecting 2 nodes represent coauthorship between the 2 authors, and thicker links represent more collaboration between the 2 authors, as shown in Figure 4.

### Reference Co-Citation Analysis

**Figure 5:**
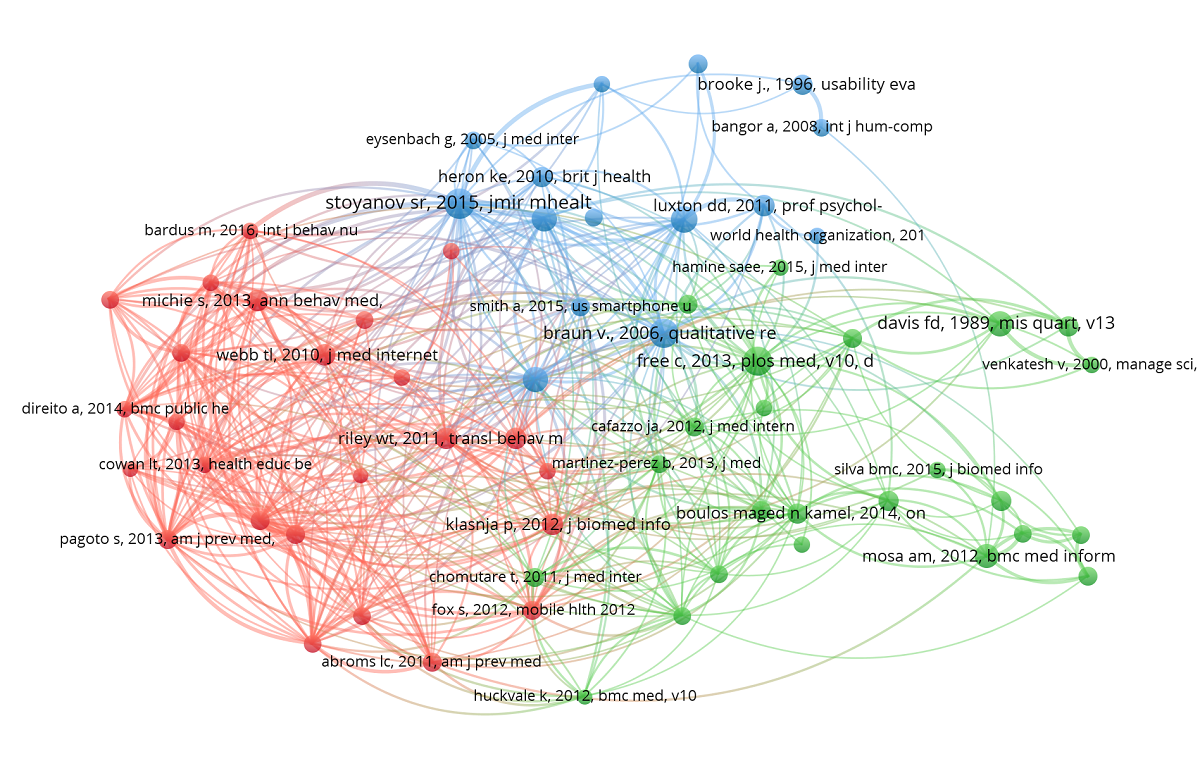
The co-citation network of references in mobile health app research, 2000-2019.

Through the co-citation analysis (examining references cited in publications), we explored the knowledge base for the mobile health apps field. We identified 2802 mobile health app publications, which cited 76,721 references, averaging 27 references per publication. The top 10 most frequently cited references are listed in Table 4. The publication that received the most citations, Stoyanov and colleagues’ “Mobile App Rating Scale: A New Tool for Assessing the Quality of Health Mobile Apps,” was published in *JMIR mHealth and uHealth* in 2015 and received a total of 106 citations as of October 6, 2019. We chose the top 64 references, which were cited at least 30 times by the retrieved papers, to generate a visualization network map with VOSviewer of co-cited references in mobile health app research. This visualization network showed 3 main clusters marked in different colors, as shown in Figure 5.

**Table 4:** Top 10 cited references in mobile health app research, 2000-2019.

Rank	Author	Journal	Title	Citations, n
1	Stoyanov SR et al (2015)	*JMIR mHealth and uHealth*	Mobile App Rating Scale: A New Tool for Assessing the Quality of Health Mobile Apps	106
2	Free C et al (2013)	*Public Library of Science Medicine*	The Effectiveness of Mobile-Health Technologies to Improve Health Care Service Delivery Processes: A Systematic Review and Meta-Analysis	101
3	Braun V and Clarke V (2006)	*Qualitative Research in Psychology*	Using Thematic Analysis in Psychology	98
4	Donker T et al (2013)	*Journal of Medical Internet Research*	Smartphones for Smarter Delivery of Mental Health Programs: A Systematic Review	85
5	Krebs P and Duncan DT(2015)	*JMIR mHealth and uHealth*	Health App Use Among US Mobile Phone Owners: A National Survey	79
6	Davis FD (1989)	*Management Information Systems Quarterly*	Perceived Usefulness, Perceived Ease of Use, and User Acceptance of Information Technology	78
7	Dennison L et al (2013)	*Journal of Medical Internet Research*	Opportunities and Challenges for Smartphone Applications in Supporting Health Behavior Change: Qualitative Study	77
8	Mosa AM et al (2012)	*BMC Medical Informatics and Decision Making*	A Systematic Review of Healthcare Applications for Smartphones	71
9	Luxton DD et al (2011)	*Professional Psychology: Research and Practice*	mHealth for Mental Health: Integrating Smartphone Technology in Behavioral Healthcare	58
10	Riley WT et al (2011)	*Translational Behavioral Medicine*	Health Behavior Models in the Age of Mobile Interventions	58

### Co-Occurrence Analysis of Top 100 Keywords

Keywords cover the main topics of a publication and are well suited to be used for analyzing related research hot spots. The research hot spots of mobile health app research were identified through co-occurrence analysis of the top 100 keywords. We used VOSviewer to extract and cluster the top 100 keywords. Multimedia Appendix 1 shows the frequency and clustering of the top 100 keywords.

**Figure 6:**
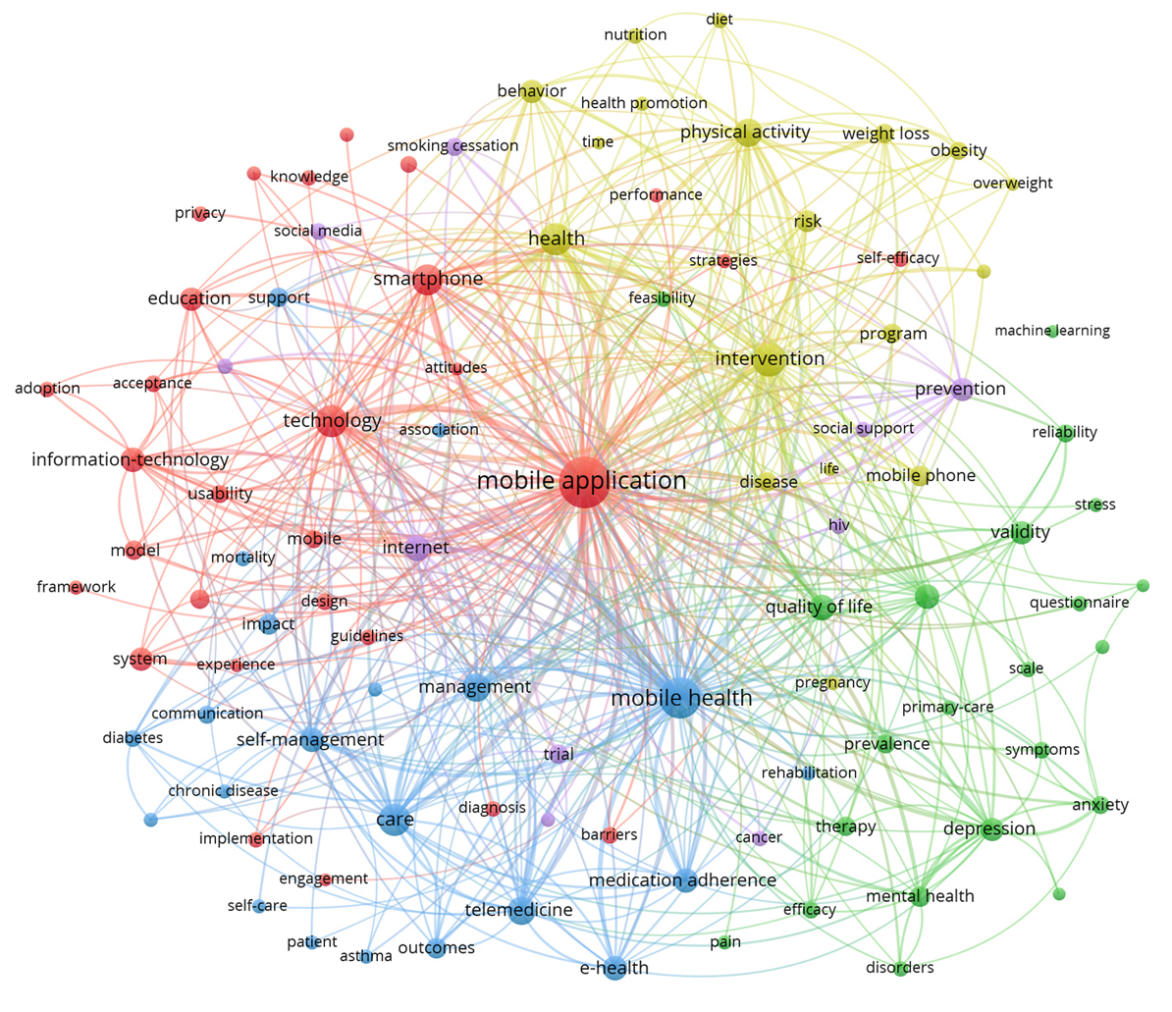
The co-occurrence network of the top 100 keywords in mobile health app research, 2000-2019.

As shown in Figure 6, we used VOSviewer to build a visualization network map of the top 100 keywords in 5 clusters with their co-occurrence. The keywords mobile application (1124), mobile health (631), intervention (347), health (329), and technology (315) are located at the center of the visualization network map. The 5 clusters are represented by color: red (cluster 1), green (cluster 2), blue (cluster 3), yellow (cluster 4), and purple (cluster 5). The node label is the keyword, and the node size represents the frequency of the keywords. Links connecting 2 nodes represent a co-occurrence relationship between the keywords. For visualization purposes, the top 500 links among keywords were determined based on how frequently they co-occur in the same publication.

## Discussion

In the current study, we explored the bibliometric characteristics of mobile health app research and we identified research trends, research hot spots, and the knowledge base associated with mobile health apps through our co-word analysis of the top 100 keywords.

### Global Trends in Mobile Health App Research

The change in the number of academic publications in a field is an important indicator of the evolving trends in this field. Mobile health app research included over 2800 publications around the world. The number of research papers published on mobile health apps annually has been increasing since 2000, with particularly notable gains in the past 5 years.

For journal sources, the top 3 journals publishing mobile health app research belong to the area of medical informatics, accounting for 18.24% (511/2802) of the total publications. Meanwhile, 848 journals had only 1 mobile health app–related publication, accounting for 30.26% (848/2802) of the total publications. Journals publishing mobile health app research were widely distributed across the general health domain, with higher concentration, as expected, in the field of medical informatics.

### The Coauthorship Networks in Mobile Health App Research

Due to the accessibility of bibliometric analysis, coauthorship is frequently used as a proxy for research collaboration. A coauthorship network can reflect the collaborative relationship among researchers and provide potential opportunities for other researchers to cooperate; examining this network can highlight potential opportunities for enhanced collaboration both within and outside of the existing network. The coauthorship network reflects author, institution, and country.

We found that the United States was the most significant contributor to mobile health app research. Of the publications identified, 65.06% (1823/2802) were published in the United States, England, Australia, and Canada, the current global leaders in mobile health app research. Figure 2 shows that the United States is the center of an international coauthorship network and cooperates with many countries/regions. The yellow coauthorship networks are mainly Asian countries/regions, such as China, Korea, and Singapore. The blue coauthorship networks are dominated by European countries such as Germany, Spain, and Italy. Our findings indicate that cooperation among countries/regions has certain regional characteristics. These groupings prompt speculation that cooperation on mobile health app research among countries/regions may be influenced by geographic proximity or by a shared language.

We found that 7 of the top 10 most productive institutions are from the United States, and the other 3 are from Canada, Australia, and South Korea. Figure 3 shows that the top 10 institutions are almost at the core of each coauthorship network. These most productive institutions and groups are leading the trends in mobile health app research. There is an excellent cooperative relationship between these institutions.

We identified 13,040 authors who have published research on mobile health apps. Of those authors, only 233 (1.79%) have published more than 4 papers in this emerging field, forming 5 relatively small coauthorship networks. We conclude from this finding that there are many researchers interested in pursuing mobile health app research, but collaboration between authors remains limited. Promoting collaboration between authors, institutions, and countries would expand the number of authors regularly publishing in this field and could contribute to more effective dissemination of innovative practices in mobile health app use.

### Basic Knowledge and Hot Topics in Mobile Health Apps

More than three-quarters of the top 10 most frequently cited references in mobile health app research were published after 2011. This timeline is consistent with our understanding of mobile health app research as a rapidly emerging field of study. As shown in Figure 5, the most frequently cited references formed 3 clusters (shown in red, green, and blue). These clusters correspond to 3 basic groupings of research: (1) promotion of health behavior change (in red), (2) evaluation of quality of mobile health apps (in green), and (3) assessment of efficiency of mobile health apps (in blue).

Keywords are standardized terms used to ensure that publications are indexed uniformly by topic. Therefore, mapping the co-word network by analyzing the co-occurrence frequency of keywords from multiple publications is helpful to study the internal structure and the hot topics in the field of mobile health app research [21]. As shown in Figure 6, there were 5 clusters of mobile health app research that were formed by co-occurrence cluster analysis of the top 100 keywords. Combined with the characteristics of mobile health apps, the 5 clusters were analyzed as described below.

Cluster 1 (red cluster) mainly focuses on the technology and system development of mobile health apps and includes 29 high-frequency keywords, such as mobile app, technology, smartphone, system, model, usability, acceptance, design, devices, barriers, privacy, and attitudes. With the continuous development of smartphone and information technology, researchers need to update or develop new mobile health apps to meet the growing needs of patients and medical staff. The system development of mobile health apps mainly includes a user-computer interface, algorithms, privacy, design, and computer security, and it follows the principles of user-centered, convenient operation, safety, and stability [22,23]. As a new product, the effectiveness, quality, and accuracy of various mobile health apps used in health care need to be continuously evaluated through academic research [24,25].

Cluster 2 (green cluster) mainly focuses on mobile health apps used in mental health and includes 22 high-frequency keywords, such as quality of life, depression, validity, mental health, prevalence, therapy, anxiety, reliability, efficacy, disorders, questionnaire, stress, and cognitive behavioral therapy. It is reported that about 29% of humans suffer from mental illness in their lifetime, and more than 55% of these do not receive the treatment they need [26]. Mobile health apps can provide instant support, anonymity, customization, and low cost. These characteristics can potentially improve access to mental health services, thereby improving the equity of mental health resource allocation. Mobile health apps can be used as independent self-help mental health assessment tools and can be used to deliver online interventions aimed at diagnosis, treatment, and monitoring. Importantly, mobile health apps improve the accessibility of treatment through ecological momentary assessment to reduce the barriers to face-to-face help, especially in patients with depression, anxiety, stress, and other symptoms [27,28].

Cluster 3 (blue cluster) focuses on mobile health apps used as mobile health tools in telemedicine, chronic disease, and medication adherence management and includes 21 high-frequency keywords such as mobile health, care, telemedicine, self-management, eHealth, medication adherence, communication, diabetes, chronic disease, glycemic control, hypertension, and asthma. Telemedicine delivered using mobile health apps is an innovative model of health care, with significant potential to solve challenges in today's health care environment [29]. This approach can provide cost-effective solutions that bridge geographical and institutional barriers [30]. Mobile health app use for telemedicine is gaining in popularity in developing countries, where medical institutions are often remote and inaccessible [31,32]. Globally, chronic diseases currently account for 60% of the global disease burden [33]. Importantly, patients with chronic diseases are prone to secondary complications, which can be prevented by strengthening patient education and self-management. Mobile health apps can be used in a variety of environments, enhancing their effectiveness as tools for self-management and monitoring [34]. They are widely used in the management of diabetes, hypertension, and asthma [35,36]. Mobile health apps can provide personalized medication adherence reminders and early warnings and improve medication adherence in patients [37,38].

Cluster 4 (yellow cluster) mainly focuses on mobile health apps used in health behavior and health promotion and includes 18 high-frequency keywords, such as intervention, health, physical activity, behavior, risk, weight loss, obesity, nutrition, diet, health promotion, and overweight. With the development of portable wearable devices and smart sensors, mobile health apps can provide self-tracking capabilities. People can track measures of interest such as weight, calories consumed, heart rate, respiratory rate, and exercise status, and can also record how they feel or how they are responding to treatment (eg, side effects). Tracking capabilities of this type can be used by people to promote the adoption of healthful behaviors, such as physical exercise, reasonable diet, and obesity prevention [39-41].

Cluster 5 (purple cluster) mainly focuses on mobile health apps used in disease prevention via the internet and includes 10 high-frequency keywords, such as internet, prevention, trial, smoking cessation, social media, cancer, and human immunodeficiency virus (HIV). The International Telecommunication Union estimates that 4.1 billion people were using the internet at the end of 2019 [42]. The internet can provide anonymity, low intervention costs, and the ability to fulfill effective solutions. Building on these characteristics, internet-based mobile health apps can promote disease prevention to solve health problems. Compared with traditional disease prevention, mobile health apps using social media technology can attract users in a more interactive way, provide convenient health education and rapid internet intervention, and achieve excellent results in HIV, smoking cessation, and cancer [43-45].

### Limitations

Our study is, to our knowledge, the first bibliometric analysis of mobile health app–related publications. Still, there are some limitations to this study. First, there may be language bias because, although we did not place any limits on the language of publications in our study, most WOS publications are in English. Second, the quality of publications in WOS is not uniform. Conducting a weighted analysis of publications based on the assessment of quality was outside the scope of our study; therefore, it is possible that our analysis has given equal attention to publications of differing quality. Finally, the current data for analysis were only extracted from WOS, excluding data extracted from other search engines such as Scopus (Elsevier), PubMed, or Google Scholar (Google LLC). Thus, it is possible that publications appearing only through one of these other search engines have been missed. We plan to address this by exploring ways of combining different data sources in future work.

### Conclusions

Through the bibliometric quantitative analysis and visualization network map of the data extracted from the WOS database, the current study reveals the research status, research trends, hot spots, and coauthorship network of mobile health app research. Mobile health app research is a new and promising field globally, with great potential for improving patient care and promoting health. By comprehensively summarizing the trends in mobile health app research, we expect this work may serve as a guide for facilitating future research directions to advance this field of research further.

## Appendix

Multimedia Appendix 1 Top 100 keywords and 5 clusters in mobile health app research, 2000-2019.

## Abbreviations

HIV: human immunodeficiency virus
WOS: Web of Science

## References

[1] Zhao J, Freeman B, Li M (2016). Can Mobile Phone Apps Influence People's Health Behavior Change? An Evidence Review. J Med Internet Res.

[2] Larson RS (2018). A Path to Better-Quality mHealth Apps. JMIR Mhealth Uhealth.

[3] Zion Market Research.

[4] Martínez-Pérez B, de LTI, López-Coronado M (2013). Mobile health applications for the most prevalent conditions by the World Health Organization: review and analysis. J Med Internet Res.

[5] Guler AT, Waaijer CJF, Palmblad M (2016). Scientific workflows for bibliometrics. Scientometrics.

[6] Ahmadvand A, Kavanagh D, Clark M, Drennan J, Nissen L (2019). Trends and Visibility of "Digital Health" as a Keyword in Articles by JMIR Publications in the New Millennium: Bibliographic-Bibliometric Analysis. J Med Internet Res.

[7] Taj F, Klein MCA, van Halteren A (2019). Digital Health Behavior Change Technology: Bibliometric and Scoping Review of Two Decades of Research. JMIR Mhealth Uhealth.

[8] Zou L, Sun L (2019). Global diabetic kidney disease research from 2000 to 2017: A bibliometric analysis. Medicine (Baltimore).

[9] Agarwal A, Durairajanayagam D, Tatagari S, Esteves S, Harlev A, Henkel R, Roychoudhury S, Homa S, Puchalt N, Ramasamy R, Majzoub A, Ly K, Tvrda E, Assidi M, Kesari K, Sharma R, Banihani S, Ko E, Abu-Elmagd M, Gosalvez J, Bashiri A (2016). Bibliometrics: tracking research impact by selecting the appropriate metrics. Asian J Androl.

[10] Li F, Li M, Guan P, Ma S, Cui L (2015). Mapping Publication Trends and Identifying Hot Spots of Research on Internet Health Information Seeking Behavior: A Quantitative and Co-Word Biclustering Analysis. J Med Internet Res.

[11] Cozzens SE, Callon M, Law J, Rip A (1988). Mapping the Dynamics of Science and Technology: Sociology of Science in the Real World. Contemporary Sociology.

[12] Zhang C, Yu Q, Fan Q, Duan Z (2013). Research collaboration in health management research communities. BMC Med Inform Decis Mak.

[13] Zhang J, Xie J, Hou W, Tu X, Xu J, Song F, Wang Z, Lu Z (2012). Mapping the Knowledge Structure of Research on Patient Adherence: Knowledge Domain Visualization Based Co-Word Analysis and Social Network Analysis. PLoS ONE.

[14] Wang Y, Wang Q, Wei X, Shao J, Zhao J, Zhang Z, Chen Z, Bai Y, Wang N, Wang Y, Li M, Zhai X (2017). Global scientific trends on exosome research during 2007–2016: a bibliometric analysis. Oncotarget.

[15] Zhao J, Yu G, Cai M, Lei X, Yang Y, Wang Q, Zhai X (2018). Bibliometric analysis of global scientific activity on umbilical cord mesenchymal stem cells: a swiftly expanding and shifting focus. Stem Cell Res Ther.

[16] Miao Y, Xu S, Chen L, Liang G, Pu Y, Yin L (2017). Trends of long noncoding RNA research from 2007 to 2016: a bibliometric analysis. Oncotarget.

[17] Khan MS, Ullah W, Riaz IB, Bhulani N, Manning WJ, Tridandapani S, Khosa F (2016). Top 100 cited articles in cardiovascular magnetic resonance: a bibliometric analysis. J Cardiovasc Magn Reson.

[18] Romero L, Portillo-Salido E (2019). Trends in Sigma-1 Receptor Research: A 25-Year Bibliometric Analysis. Front. Pharmacol.

[19] van Eck NJ, Waltman L (2009). Software survey: VOSviewer, a computer program for bibliometric mapping. Scientometrics.

[20] Eck NJV, Waltman L (2009). How to normalize cooccurrence data? An analysis of some well-known similarity measures. J. Am. Soc. Inf. Sci.

[21] Gan J, Cai Q, Galer P, Ma D, Chen X, Huang J, Bao S, Luo R (2019). Mapping the knowledge structure and trends of epilepsy genetics over the past decade. Medicine.

[22] Schnall R, Rojas M, Bakken S, Brown W, Carballo-Dieguez A, Carry M, Gelaude D, Mosley JP, Travers J (2016). A user-centered model for designing consumer mobile health (mHealth) applications (apps). J Biomed Inform.

[23] Martínez-Pérez B, de LTI, López-Coronado M (2015). Privacy and security in mobile health apps: a review and recommendations. J Med Syst.

[24] Singler K, Roth T, Beck S, Cunningham M, Gosch M (2015). Development and initial evaluation of a point-of-care educational app on medical topics in orthogeriatrics. Arch Orthop Trauma Surg.

[25] Vagge A, Wangtiraumnuay N, Pellegrini M, Scotto R, Iester M, Traverso CE (2019). Evaluation of a Free Public Smartphone Application to Detect Leukocoria in High-Risk Children Aged 1 to 6 Years. J Pediatr Ophthalmol Strabismus.

[26] Anthes E (2016). Mental health: There's an app for that. Nature.

[27] Donker T, Petrie K, Proudfoot J, Clarke J, Birch M, Christensen H (2013). Smartphones for smarter delivery of mental health programs: a systematic review. J Med Internet Res.

[28] Lemey C, Larsen ME, Devylder J, Courtet P, Billot R, Lenca P, Walter M, Baca-García E, Berrouiguet S (2019). Clinicians’ Concerns About Mobile Ecological Momentary Assessment Tools Designed for Emerging Psychiatric Problems: Prospective Acceptability Assessment of the MEmind App. J Med Internet Res.

[29] Bashshur RL, Howell JD, Krupinski EA, Harms KM, Bashshur N, Doarn CR (2016). The Empirical Foundations of Telemedicine Interventions in Primary Care. Telemed J E Health.

[30] Silva BMC, Rodrigues JJPC, de LTDI, López-Coronado M, Saleem K (2015). Mobile-health: A review of current state in 2015. J Biomed Inform.

[31] Déglise C, Suggs LS, Odermatt P (2012). Short Message Service (SMS) Applications for Disease Prevention in Developing Countries. J Med Internet Res.

[32] Källander K, Tibenderana JK, Akpogheneta OJ, Strachan DL, Hill Z, ten Asbroek AHA, Conteh L, Kirkwood BR, Meek SR (2013). Mobile Health (mHealth) Approaches and Lessons for Increased Performance and Retention of Community Health Workers in Low- and Middle-Income Countries: A Review. J Med Internet Res.

[33] World Health Organization (2002). World Health Organization.

[34] Parmanto B, Pramana G, Yu DX, Fairman AD, Dicianno BE, McCue MP (2013). iMHere: A Novel mHealth System for Supporting Self-Care in Management of Complex and Chronic Conditions. JMIR Mhealth Uhealth.

[35] Lum E, Jimenez G, Huang Z, Thai L, Semwal M, Boehm BO, Car J (2019). Decision Support and Alerts of Apps for Self-management of Blood Glucose for Type 2 Diabetes. JAMA.

[36] Holmen H, Torbjørnsen A, Wahl AK, Jenum AK, Småstuen MC, Arsand E, Ribu L (2014). A Mobile Health Intervention for Self-Management and Lifestyle Change for Persons With Type 2 Diabetes, Part 2: One-Year Results From the Norwegian Randomized Controlled Trial RENEWING HEALTH. JMIR Mhealth Uhealth.

[37] Zanetti-Yabur A, Rizzo A, Hayde N, Watkins AC, Rocca JP, Graham JA (2017). Exploring the usage of a mobile phone application in transplanted patients to encourage medication compliance and education. The American Journal of Surgery.

[38] Labovitz DL, Shafner L, Reyes GM, Virmani D, Hanina A (2017). Using Artificial Intelligence to Reduce the Risk of Nonadherence in Patients on Anticoagulation Therapy. Stroke.

[39] Stavrositu C, Kim J (2018). Self-Persuasion Through Mobile Applications: Exploring Different Routes to Health Behavioral Change. Cyberpsychology, Behavior, and Social Networking.

[40] Dennison L, Morrison L, Conway G, Yardley L (2013). Opportunities and challenges for smartphone applications in supporting health behavior change: qualitative study. J Med Internet Res.

[41] Martin C, Gilmore L, Apolzan J, Myers C, Thomas D, Redman L (2016). Smartloss: A Personalized Mobile Health Intervention for Weight Management and Health Promotion. JMIR Mhealth Uhealth.

[42] ITUPublications.

[43] Schnall R, Mosley JP, Iribarren SJ, Bakken S, Carballo-Diéguez A, Brown IW (2015). Comparison of a User-Centered Design, Self-Management App to Existing mHealth Apps for Persons Living With HIV. JMIR Mhealth Uhealth.

[44] Powell AC, Torous J, Chan S, Raynor GS, Shwarts E, Shanahan M, Landman AB (2016). Interrater Reliability of mHealth App Rating Measures: Analysis of Top Depression and Smoking Cessation Apps. JMIR Mhealth Uhealth.

[45] Coughlin S, Thind H, Liu B, Champagne N, Jacobs M, Massey RI (2016). Mobile Phone Apps for Preventing Cancer Through Educational and Behavioral Interventions: State of the Art and Remaining Challenges. JMIR Mhealth Uhealth.

