# Supplementary material for: Theme Trends and Knowledge Structure on Mobile Health Apps: Bibliometric Analysis
Source: JMIR Mhealth Uhealth. 2020 Jul 27;8(7):e18212. doi: 10.2196/18212 (PMC7418015; doi:10.2196/18212)
Supplement: Multimedia Appendix 1 [file mhealth_v8i7e18212_app1.docx]

Multimedia Appendix 1. Top 100 keywords and 5 clusters in mobile health app research, 2000-2019.

| Cluster | Keywords | Frequency, n | Rank |
| --- | --- | --- | --- |
| 1 | mobile application | 1124 | 1 |
| 1 | technology | 315 | 5 |
| 1 | smartphone | 277 | 7 |
| 1 | information-technology | 146 | 15 |
| 1 | education | 130 | 21 |
| 1 | system | 126 | 22 |
| 1 | model | 84 | 29 |
| 1 | health-care | 76 | 31 |
| 1 | mobile | 70 | 38 |
| 1 | usability | 70 | 40 |
| 1 | acceptance | 51 | 49 |
| 1 | mobile technology | 51 | 50 |
| 1 | barriers | 50 | 51 |
| 1 | design | 49 | 52 |
| 1 | diagnosis | 43 | 58 |
| 1 | adoption | 41 | 61 |
| 1 | guidelines | 41 | 62 |
| 1 | privacy | 41 | 64 |
| 1 | implementation | 40 | 67 |
| 1 | knowledge | 40 | 68 |
| 1 | self-efficacy | 39 | 70 |
| 1 | performance | 38 | 72 |
| 1 | devices | 37 | 73 |
| 1 | perceptions | 35 | 78 |
| 1 | strategies | 32 | 88 |
| 1 | framework | 31 | 90 |
| 1 | attitudes | 30 | 92 |
| 1 | engagement | 28 | 95 |
| 1 | experience | 28 | 96 |
| 2 | quality of life | 175 | 12 |
| 2 | randomized controlled trial | 142 | 17 |
| 2 | depression | 137 | 18 |
| 2 | validity | 133 | 19 |
| 2 | mental health | 97 | 25 |
| 2 | prevalence | 73 | 33 |
| 2 | therapy | 72 | 37 |
| 2 | anxiety | 69 | 41 |
| 2 | reliability | 60 | 44 |
| 2 | efficacy | 59 | 45 |
| 2 | symptoms | 54 | 48 |
| 2 | scale | 46 | 54 |
| 2 | disorders | 45 | 55 |
| 2 | feasibility | 44 | 56 |
| 2 | questionnaire | 41 | 65 |
| 2 | primary-care | 34 | 80 |
| 2 | stress | 33 | 83 |
| 2 | ecological momentary assessment | 31 | 89 |
| 2 | pain | 31 | 91 |
| 2 | cognitive-behavioral therapy | 30 | 93 |
| 2 | machine learning | 27 | 99 |
| 2 | mindfulness | 26 | 100 |
| 3 | mobile health | 631 | 2 |
| 3 | care | 299 | 6 |
| 3 | telemedicine | 221 | 8 |
| 3 | management | 209 | 10 |
| 3 | self-management | 148 | 13 |
| 3 | e-health | 147 | 14 |
| 3 | medication adherence | 143 | 16 |
| 3 | outcomes | 96 | 26 |
| 3 | impact | 87 | 28 |
| 3 | support | 72 | 36 |
| 3 | communication | 60 | 43 |
| 3 | diabetes | 58 | 47 |
| 3 | mortality | 41 | 63 |
| 3 | chronic disease | 36 | 74 |
| 3 | glycemic control | 35 | 76 |
| 3 | hypertension | 33 | 81 |
| 3 | rehabilitation | 33 | 82 |
| 3 | association | 32 | 84 |
| 3 | asthma | 32 | 85 |
| 3 | patient | 32 | 86 |
| 3 | self-care | 32 | 87 |
| 4 | intervention | 347 | 3 |
| 4 | health | 329 | 4 |
| 4 | physical activity | 213 | 9 |
| 4 | behavior | 120 | 23 |
| 4 | risk | 111 | 24 |
| 4 | mobile phone | 88 | 27 |
| 4 | weight loss | 82 | 30 |
| 4 | disease | 72 | 34 |
| 4 | program | 72 | 35 |
| 4 | obesity | 70 | 39 |
| 4 | nutrition | 42 | 60 |
| 4 | diet | 40 | 66 |
| 4 | health promotion | 38 | 71 |
| 4 | health behavior | 35 | 77 |
| 4 | pregnancy | 34 | 79 |
| 4 | overweight | 29 | 94 |
| 4 | time | 28 | 97 |
| 4 | life | 27 | 98 |
| 5 | internet | 176 | 11 |
| 5 | prevention | 131 | 20 |
| 5 | trial | 76 | 32 |
| 5 | smoking cessation | 61 | 42 |
| 5 | social media | 59 | 46 |
| 5 | cancer | 46 | 53 |
| 5 | HIV^a^ | 44 | 57 |
| 5 | qualitative research | 43 | 59 |
| 5 | social support | 40 | 69 |
| 5 | digital health | 36 | 75 |

^a^HIV: human immunodeficiency virus.
